# Functional advantages of triplication of the 3B coding region of the FMDV genome

**DOI:** 10.1096/fj.202001473RR

**Published:** 2020-11-23

**Authors:** Oluwapelumi O. Adeyemi, Joseph C. Ward, Joseph S. Snowden, Morgan R. Herod, David J. Rowlands, Nicola J. Stonehouse

**Affiliations:** ^1^ School of Molecular and Cellular Biology Faculty of Biological Sciences and Astbury Centre for Structural Molecular Biology University of Leeds Leeds UK; ^2^ Department of Medical Microbiology and Parasitology Faculty of Basic Medical Sciences College of Health Sciences University of Ilorin Ilorin Nigeria

**Keywords:** 3B, evolution, FMDV, gene duplication, picornavirus, trans‐complementation

## Abstract

For gene duplication to be maintained, particularly in the small genomes of RNA viruses, this should offer some advantages. We have investigated the functions of a small protein termed VPg or 3B, which acts as a primer in the replication of foot‐and‐mouth disease virus (FMDV). Many related picornaviruses encode a single copy but uniquely the FMDV genome includes three (nonidentical) copies of the 3B coding region. Using sub‐genomic replicons incorporating nonfunctional 3Bs and 3B fusion products in competition and complementation assays, we investigated the contributions of individual 3Bs to replication and the structural requirements for functionality. We showed that a free N‐terminus is required for 3B to function as a primer and although a single 3B can support genome replication, additional copies provide a competitive advantage. However, a fourth copy confers no further advantage. Furthermore, we find that a minimum of two 3Bs is necessary for *trans* replication of FMDV replicons, which is unlike other picornaviruses where a single 3B can be used for both *cis* and *trans* replication. Our data are consistent with a model in which 3B copy number expansion within the FMDV genome has allowed evolution of separate *cis* and *trans* acting functions, providing selective pressure to maintain multiple copies of 3B.

**Abbreviations**
ATCC: American type culture collection
BHK‐21: baby hamster kidney cell‐lot 21
cDNA: complementary deoxyribonucleic acid
DMEM: Dulbecco’s minimum essential media
EV71: enterovirus‐71
FBS: fetal bovine serum
FMDV: foot‐and‐mouth disease virus
h.p.t.: hours post‐transfection
kb: kilobase
MUSCLE: Multiple Sequence Comparison by Log‐Expectation
NCBI: National Center for Biotechnology Information
NEB: New England Biolabs
ORF: open reading frame
PBS: phosphate‐buffered saline
pol: polymerase
pRep: plasmid replicon
pro: protease
PV: poliovirus
RdRp: RNA‐dependent RNA polymerase
RNA: ribonucleic acid
S.D.: standard deviation
S.E.M.: standard error of the mean
SDS‐PAGE: sodium dodecyl sulfate poly‐acrylamide gel electrophoresis
T_N_T: transcription and translation
VPg: genome‐linked viral protein
WT: wild‐type
Y3F: tyrosine to phenylalanine substitution of the third residue in the protein

## 1 INTRODUCTION

The evolution of small RNA viruses is largely driven by the error‐prone RNA‐dependent RNA polymerase (RdRp), which is responsible for replicating the viral genome. Hence, RNA viruses exist as populations of related quasispecies.^1^ Virus quasispecies undergo continuous genetic variation, shaped by selection, and counterbalanced by complementation.^1^, ^2^ Complementation between quasispecies members can compensate for the presence of genomes defective by point mutations or truncation.^3^ Furthermore, recombination events can result in gene duplication,^4^ which can facilitate the evolution of novel genes (reviewed in^5^). However, while gene duplication is common among DNA viruses,^6^ it is rarely reported among RNA viruses, although it should be noted that the capsid proteins of picornaviruses probably evolved via this mechanism.^7^

Foot‐and‐mouth disease (FMD) is a highly contagious disease affecting cloven‐hoofed animals, which imposes a global economic burden of between US$ 6.5 and 21 billion in endemic regions, and over US$ 1.5 billion annually in FMD‐free countries and regions.^8^, ^9^ The causative agent of FMD is foot‐and‐mouth disease virus (FMDV): an ∼8.5 kb single‐stranded positive‐sense RNA virus in the *Aphthovirus* genus of the *Picornaviridae* family. The FMDV genome comprises an open reading frame (ORF) flanked at the 5ʹ and 3ʹ ends by untranslated regions. The single ORF encodes both the structural and nonstructural proteins starting with the leader protease followed by a capsid coding P1‐2A region. P1 is processed into the four structural proteins VP4, VP2, VP3, and VP1. Downstream of the P1‐2A region lies the nonstructural protein‐coding region, required for RNA replication and organized as two sub‐regions, P2 and P3. The P2 region encodes the 2B and 2C proteins, 2A being a C‐terminal extension of P1. The P3 region encodes 3A, triplicate copies of the replication primer, 3B (also known as VPg, ie, 3B1, 3B2, and 3B3), the viral protease 3C^pro^ and the RdRp termed 3D^pol^.^10^

Most picornaviruses, such as the well‐studied poliovirus (PV), encode a single copy of 3B and although there is evidence for duplication of 3B in some, FMDV is the only picornavirus that has been demonstrated to have three (nonidentical) copies of the 3B coding region. This feature is almost 100% conserved among naturally occurring FMDV isolates to date.^11^ This implies a strong selection pressure in FMDV for the maintenance of three copies of 3B, for currently unknown reasons. The 3B proteins comprise 23 to 24 residues and are well conserved, with over 78% identity at the amino acid and nucleotide levels^7^, ^12^ (Figure S1). Interestingly, the Eurasian and Southern African Territories serotypes fall into distinctive 3B sequence conservation groups, reflecting their separate lineages. However, despite the genetic distance between them, all serotypes maintain three nonidentical copies of the 3B coding region (Figure S1). There is an invariant tyrosine residue at the third position of each 3B protein which is uridylylated.^12^, ^13^ This reaction is performed by a complex of the 3D^pol^, the 3CD precursor protein and a viral RNA structure termed the cis‐active replicative element or *cre*.^14^ The resulting 3B‐pUpU functions as a primer for RNA synthesis resulting in the generation of new viral strands, each with a covalently linked 3B protein at the 5ʹ end.^15^

Earlier studies, using sub‐genomic replicons and infectious virions, have shown that although multiple copies of 3B may influence host specificity and virulence,^11^ a single copy is sufficient to support replication.^16^, ^17^ Previously, using mutant FMDV replicon constructs, we showed that the sequence at the boundary between 3B3 and 3C is essential for correct processing of the polyprotein.^17^ Here, we examine differences in the functions of the individual copies of 3B, which help to explain why possession of multiple copies is advantageous to the virus, and hence, why these persist in the population.

## 2 METHODOLOGY

### 2.1 Cell lines

Baby hamster kidney cells‐lot 21 (BHK‐21) and HeLa cells were obtained from ATCC (Manassas, Virginia, USA) and propagated according to standard methods.

### 2.2 Replicon plasmid constructs

Two previously described picornaviral sub‐genomic replicons in which the capsid protein coding regions were replaced with fluorescent reporter genes mCherry^18^ or ptGFP^19^ were used for this study. The FMDV sub‐genomic replicon includes three copies of 3B^20^ and the PV sub‐genomic replicon has one copy of 3B.^21^ In addition, EV71 replicons (pRepEV71) with both reporter genes were generated, which also have a single copy of 3B. Substitutions were introduced into the sub‐genomic replicons by site‐directed mutagenesis. FMDV mutant constructs with only one copy of 3B (ie, 3B1, 3B2, or 3B3) or multiple copies of 3Bs (ie, 3B1+3, 3B2+3, 3B1+3) were designed to incorporate the natural 3A‐3B1 and 3B3‐3C junctions. Further FMDV constructs were designed with substitutions at specific cleavage boundaries within P3 to inhibit 3C^pro^ proteolysis, and hence, produce a series of defined fusion proteins. An FMDV construct was also designed to incorporate four copies of 3B by duplicating the 3B1 sequence using alternative codons (ie, 3Bx123). The duplication was inserted between 3A and 3B1, while retaining the 3A‐3B1 and 3B1‐3B2 junctions. The replicon constructs used in this study are listed in Table 1, with additional details provided in Figure S2. The sequences of primers are available on request.

**TABLE 1:** FMDV sub‐genomic replicon constructs

Construct	P3 modification
WT	Nil
3D‐GNN	Inactivating mutation to the active site of 3D^pol^ (i.e. GDD > GNN).
3B123^Y3F^	Introduced Y3F mutations on all 3Bs to prevent uridylation of all 3Bs and thereby inactivate sub‐genomic replicon.
3B1^Y3F^	Introduced Y3F mutation to 3B1, while 3B2 and 3B3 are functional.
3B2^Y3F^	Introduced Y3F mutation to 3B2, while 3B1 and 3B3 are functional.
3B3^Y3F^	Introduced Y3F mutation to 3B3, while 3B1 and 3B2 are functional.
3B12^Y3F^	Introduced Y3F mutation to 3B1 and 3B2, while 3B3 is functional.
3B23^Y3F^	Introduced Y3F mutation to 3B2 and 3B3, while 3B1 is functional.
3B13^Y3F^	Introduced Y3F mutation to 3B1 and 3B3, while 3B2 is functional.
3B1	Deleted 3B2 and 3B3 and introduced the 3B3‐3C boundary to the C‐terminus of 3B1.
3B2	Deleted 3B1 and 3B3 and introduced the 3A‐3B1 and the 3B3‐3C boundary to the N‐ and C‐terminus of 3B1, respectively.
3B3	Deleted 3B1 and 3B2 and introduced the 3A‐3B1 boundary to the N‐terminus of 3B3.
3B1+2	Deleted 3B3 and introduced the 3B3‐3C boundary to the C‐terminus of 3B2.
3B2+3	Construct has the 3B1 deleted while 3B2 possesses the 3A‐3B1 boundary at the N‐terminus.
3B1+3	Construct has the 3B2 coding sequence deleted.
3A/3B1	Introduced alanine mutations at the P1 and P2 boundary positions of the 3A‐3B1 boundary to prevent proteolytic cleavage.
3B1/3B2	Introduced alanine mutations at the P1 and P2 boundary positions of the 3B1‐3B2 junction to prevent proteolytic cleavage.
3B2/3B2	Introduced alanine mutations at the P1 and P2 boundary positions of the 3B2‐3B3 junction to prevent proteolytic cleavage.
3B3/3C	Introduced alanine mutations at the P1 and P2 boundary positions of the 3B3‐3C junction to prevent proteolytic cleavage.
3C/3D	Introduced alanine mutations at the P1 and P2 boundary positions of the 3C‐3D junction to prevent proteolytic cleavage.
3A/3B1 3B23^Y3F^	Introduced alanine mutations at the P1 and P4 boundary positions of the 3A‐3B1 junction to prevent proteolytic cleavage, and Y3F mutations to 3B2 and 3B3 to prevent uridylation.
3B1/3B2 3B3^Y3F^	Introduced alanine mutations at the P1 and P2 boundary positions of the 3B1‐3B2 junction to prevent proteolytic cleavage, and Y3F mutation to 3B3 to prevent uridylation.
3B1^Y3F^ 3B2/3B3	Introduced alanine mutations at the P1 and P2 boundary positions of the 3B2‐3B3 junction to prevent proteolytic cleavage, and Y3F mutation to 3B1 to prevent uridylation.
3B12^Y3F^ 3B3/3C	Introduced alanine mutations at the P1 and P2 boundary positions of the 3B3‐3C junction to prevent proteolytic cleavage, and Y3F mutations to 3B1 and 3B2 to prevent uridylation.
3B123^Y3F^ 3C/CD	Introduced alanine mutations at the P1 and P2 boundary positions of the 3C‐3D junction to prevent proteolytic cleavage, and Y3F mutations to all 3Bs to prevent uridylation.
Δ3B1 3B2/3B3	Deleted 3B1 and introduced the 3A‐3B1 boundary to the N‐terminus of 3B2, which was fused to 3B3 using alanine mutations at the P1 and P2 positions.
Δ3B1 3B2^Y3F^/3B3	Deleted 3B1 and introduced the 3A‐3B1 boundary to the N‐terminus of 3B2. Introduced Y3F mutation to 3B2 and alanine mutations at the P1 and P2 positions of the 3B2‐3B3 junction to prevent proteolytic cleavage.
Δ3B1 3B2/3B3^Y3F^	Deleted 3B1 and introduced the 3A‐3B1 boundary to the N‐terminus of 3B2. Introduced Y3F mutation to 3B3 and alanine mutations at the P1 and P2 positions of the 3B2‐3B3 junction to prevent proteolytic cleavage.
Δ3B1 3B2^Y3F^/3B3^Y3F^	Deleted 3B1 and introduced the 3A‐3B1 boundary to the N‐terminus of 3B2. Introduced Y3F mutation to 3B2 and 3B3, and alanine mutations at the P1 and P2 positions of the 3B2‐3B3 junction to prevent proteolytic cleavage.
3Bx	Introduced an additional 3B1 to at the N‐terminus of 3B1 such that construct possesses four copies of 3Bs while maintaining the 3A‐3B1 boundary.
3Bx 3B123^Y3F^	Introduced an additional 3B1 to at the N‐terminus of 3B1 such that construct possesses four copies of 3Bs while maintaining the 3A‐3B1 boundary. Introduced Y3F mutations to 3B1, 3B2, and 3B3 to prevent uridylation.

### 2.3 In vitro transcription

Replicon plasmid DNA was linearized with *Asc*I (NEB, Ipswich, Massachusetts, USA). RNA transcripts were generated from sub‐genomic replicon cDNAs using T7 RNA polymerase (NEB, Ipswich, Massachusetts, USA) in the presence of nucleoside triphosphates (ATP, UTP, GTP, CTP) and MnCl_2_ according to the manufacturer’s protocol and our established methods.^22^ T7 RNA transcripts were purified using a Zymogen RNA Clean and Concentrator‐25 (Zymo Research, Irvine, California USA) according to the manufacturer’s instructions. Prior to transfection, RNA quality was determined by denaturing agarose gel electrophoresis^23^ and quantified by spectrophotometry.

### 2.4 Replication assays

BHK‐21 cells were propagated in duplicate wells of a 12‐well plate according to standard methods. Cells were transfected with 1 µg of purified mCherry or GFP replicon RNA transcripts using Lipofectin reagents (Thermo Fisher Scientific, Waltham, Massachusetts, USA) according to the manufacturer’s protocol. Replication was monitored hourly for red and/or green fluorescence within an IncuCyte Zoom (Sartorius, Göttingen, Germany) as previously described.^22^ Each experiment was analyzed for both total fluorescence intensity per well and fluorescent positive cells per well. We have previously shown^22^ that the same results were observed when analyzing expression using either metric, therefore, for conciseness, only the positive cells per well data are presented here.

### 2.5 End‐point replication assays

BHK‐21 cells seeded in duplicate wells of a 6‐well plate were pretreated for 1 hour with 10 µg/mL of actinomycin D at 37°C. Pretreated cells were transfected with 4 µg of replicon RNAs, before radiolabeling with 18 µCi of [^3^H]‐U (uridine) per well at 1‐hour post‐transfection (h.p.t.). At 8 h.p.t., total RNA was harvested using guanidinium thiocyanate‐phenol‐chloroform methods^24^ and quantified by spectrophotometry. A total of 1 µg RNA extract was assayed by scintillation counting and 35 µg total RNA extract purified through 5%‐25% sucrose gradient (containing 100 mM sodium acetate, 0.1% SDS) by ultracentrifugation at 370 000*g* using an SW 55 Ti rotor for 50 minutes at room temperature. Gradient fractions were counted by scintillation for [^3^H] in a scintillation cocktail according to standard protocols.^25^

### 2.6 Replicon competition assays

Equimolar concentrations of two replicon constructs that express different fluorescent reporter genes, that is, mCherry^18^ or ptGFP,^19^ were co‐transfected into BHK‐21 cells using Lipofectin reagents (Thermo Fisher Scientific, Waltham, Massachusetts, USA) according to the manufacturer’s protocol. Replication was monitored hourly for red and green fluorescence within an IncuCyte Zoom (Sartorius, Göttingen, Germany) as previously described.^22^

### 2.7 Residue alignment

The 3B amino acid sequences of FMDV reference serotypes were sourced from the databank of the National Center for Biotechnology Information (NCBI), Bethesda, Maryland, USA^26^ and aligned using the Multiple Sequence Comparison by Log‐Expectation (MUSCLE) algorithm of CLC sequencing viewer version 7 (QIAGEN, Hilden, Germany). Sequence homology was estimated as the percentage (%) amino acid sequence similarity for the respective VPg.

### 2.8 In vitro T_N_T (rabbit reticulocyte lysate) assay

Coupled transcription/translation reactions (Promega, Madison, Wisconsin, USA) were carried out using expression plasmid constructs of FMDV WT or mutated P3 polyproteins in the presence of [^35^S] labeled methionine according to the manufacturer’s protocol. Following a 40‐minute incubation, reactions were chased with excess unlabeled methionine/cysteine with sample collection at 30‐minute intervals. Reactions were stopped by addition of 2x Laemmli buffer and boiling. Proteins were separated by 12% SDS‐PAGE and detected by autoradiography.^27^

### 2.9 Statistical analyses

This was achieved by two‐tailed unpaired Student’s *t* tests using GraphPad Prism version 7.01 for Windows (GraphPad Software, La Jolla CA). Significant differences between mutants or against WT are shown as *P*‐values <.05 (*), <.01 (**), and <.001 (***). Error bars represent standard deviation (S.D.) or standard error of the mean (S.E.M.) of multiple biological experiments, as stated.

### 2.10 Structural analysis and visualization

Atomic coordinates of 3B in complex with 3D were downloaded from the Protein Data Bank (PDB‐2D7S)^28^ and visualized using UCSF Chimera.^29^ Figures were created using UCSF ChimeraX.^30^

## 3 RESULTS

### 3.1 One functional copy of 3B is sufficient for FMDV genome replication

Although previous studies have shown that FMDV can replicate with a single copy of 3B, three copies are almost invariably present in field isolates and are required for maximal efficiency of virus growth.^16^ However, the functional advantage of the triplication of 3B coding sequences found in the FMDV genome is not understood.

**FIGURE 1:**
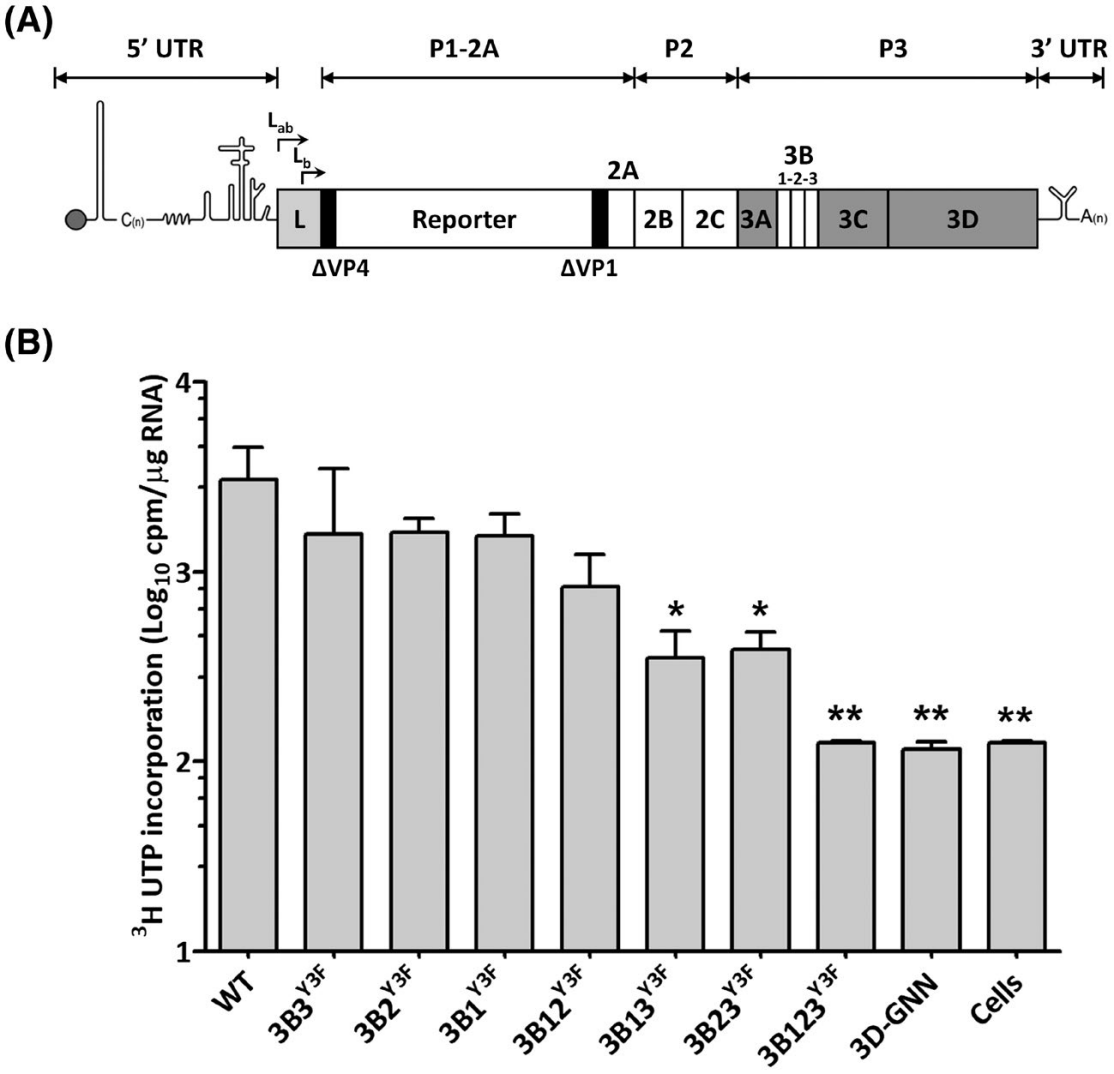
FMDV genome replication can occur with fewer than three functional copies of 3B. A, Annotated cartoon of FMDV replicon showing the capsid‐coding region replaced with a reporter gene. B, BHK‐21 cells in duplicate wells of a 6‐well plate were pretreated for 1 hour with 10 µg/mL of actinomycin D at 37°C. Cells were then transfected with T7 RNA transcripts of respective replicon constructs and radio‐labeled with [^3^H]‐U. These include WT, a polymerase active site mutant (3D‐GNN) and replicons where one, two, or all the 3Bs have been inactivated by Y3F substitution (Table 1). At 8 h.p.t., total RNA extract was harvested and quantified spectrophotometrically. RNA synthesis was quantified as scintillation counts per microgram of RNA (n = 3 ± S.D., *P < .05, **P < .01, ***P < .001)

To address the question of why FMDV maintains three copies of 3B, we first determined the efficacy of each 3B protein in priming genome replication using a sub‐genomic replicon^20^ in which the viral structural proteins were replaced with a fluorescent reporter gene (Figure 1A). To investigate the roles of individual copies of 3B on replication priming, we avoided 3B deletions and instead introduced tyrosine to phenylalanine substitutions to the third amino acid (Y3F). This substitution abrogates 3B uridylylation, thus, rendering the protein unable to function as a primer for RNA replication while minimizing structural changes to the viral genome. Replicon constructs were generated with Y3F substitutions in each copy of 3B individually or in pairs in every combination. As a control, all copies of 3B were mutated. RNA transcripts were generated for these seven replicons, a wild‐type (WT) control replicon and a replicon containing a replication‐inactivating substitution in 3D^pol^ (3D‐GNN), which served as a control for translation from the input RNA. RNA transcripts were used to transfect BHK‐21 cells and newly synthesized RNA measured by incorporation of radio‐labeled [^3^H]‐U. To maximize the labeling of replicon RNA, BHK‐21 cells were pretreated with actinomycin‐D to reduce incorporation of [^3^H]‐U into cellular RNA. After the addition of [^3^H]‐U at 1 h.p.t, total cellular RNA was harvested at 8 h.p.t., separated by sucrose gradient centrifugation and analyzed by scintillation counting (Figures 1B and S3). The replicons with two copies of functional 3B in any combination (ie, 3B1^Y3F^, 3B2^Y3F^, or 3B3^Y3F^) and the replicon with a single functional 3B3 (ie, 3B12^Y3F^) had slightly reduced levels of [^3^H]‐U incorporation, although the differences were not statistically different from WT. The replicons containing only a functional 3B1 or 3B2 (ie, 3B23^Y3F^ and 3B13^Y3F^) had nine‐ and eightfold reduction in [^3^H]‐U incorporation compared to WT, respectively. Incorporation of [^3^H]‐U from a replicon with a replication‐defective 3D‐GNN substitution was used to measure levels of input translation and the levels recorded here were similar to cells alone or to the construct lacking all three 3Bs (3B123^Y3F^).

We also monitored replication over time by measuring mCherry fluorescence using our established protocols.^22^ Replication is shown as the number of mCherry positive cells up to 8 h.p.t., after which replication plateaus (Figure S4). As we have previously described,^22^ there is no difference in the data when plotted as either total fluorescence or number of positive cells, therefore, for conciseness the latter data is shown. At 3 h.p.t., there was no significant difference in the replication of all the constructs which had two functional copies of 3B (ie, 3B1^Y3F^, 3B2^Y3F^, or 3B3^Y3F^) and these all replicated similarly to WT (Figure S4). Furthermore, there were no significant differences between replication of WT and a double mutant construct with a single functional 3B3 (ie, 3B12^Y3F^). However, constructs lacking a functional 3B3 (ie, 3B23^Y3F^ and 3B13^Y3F^) appeared to replicate less well, although this was only statistically significant for the former. Reporter expression from a construct with a replication‐defective 3D‐GNN substitution was used to measure levels of input translation. Although levels of reporter expression recorded were low as expected, it is interesting to note that for the construct lacking any active 3Bs (ie, 3B123^Y3F^), expression was slightly higher than for the 3D‐GNN control for unknown reasons.

These data are broadly consistent with those shown in Figure 1B and suggest that the presence of more than one copy of 3B confers a replication advantage and imply that 3B3 is especially important for genome priming.

### 3.2 Multiple copies of 3B provide a competitive replication advantage

**FIGURE 2:**
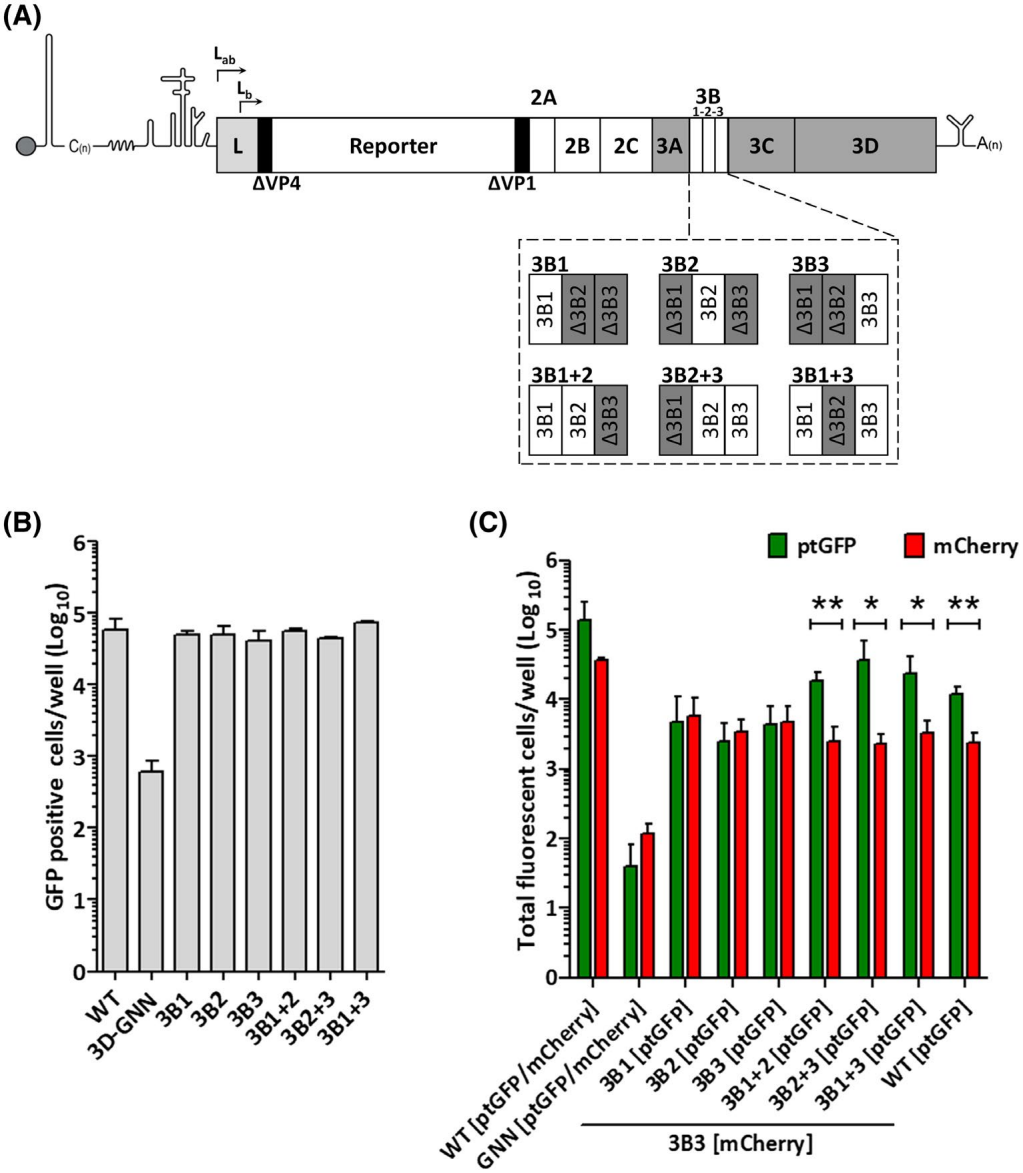
Competitive advantage of multiple copies of FMDV 3B. A, FMDV replicon, highlighting the P3 region. Constructs were designed (listed in Tables 1 and S1) with one or two copies of 3B but maintaining the 3A‐3B1 and 3B3‐3C junctions. B, BHK‐21 cells were transfected with T7 RNA transcripts of GFP replicon constructs. Expression was monitored hourly and is shown as fluorescence positive cells at 8 h.p.t. Each data set represents an average of two wells (n = 3 ± S.D.). C, Co‐transfection of BHK‐21 cells with equimolar concentrations of T7 transcripts of FMDV mCherry replicon 3B3 and ptGFP replicon constructs with various copies of 3B. Expression of mCherry and ptGFP was monitored hourly and is shown as fluorescence positive cells at 8 h.p.t. Each replicate represents an average of two wells (n = 3 ± S.D., *P < .05, **P < .01)

We speculated that the effect of multiple copies of 3B in the genome could become more apparent in a competitive environment. Therefore, to investigate any such competitive advantage, we co‐transfected cells with different combinations of replicons expressing different fluorescent reporters. FMDV replicons containing only one copy of each 3B or two copies were designed (Figure 2A). As polyprotein cleavage is mediated by the 3C^pro^ enzyme which recognizes defined boundary sequences, the natural 3A‐3B1 and 3B3‐3C junction sequences were maintained in all cases to minimize effects due to differential processing (Figure S2).

All the constructs were replication‐competent, that is, achieved WT levels of reporter gene expression at 8 h.p.t. (Figure 2B). From the data presented in Figure 1, comparing replicons with a single active 3B protein, it appeared that replication was higher for replicons that included a functional 3B3. Therefore, a mCherry replicon encoding 3B3 was selected for the competition experiments here and RNA was co‐transfected with RNA from a panel of replicons expressing ptGFP and various numbers and combinations of 3B. As controls, co‐transfections were also performed with a WT replicon, the replication‐deficient 3D‐GNN replicon or yeast tRNA (to control for transfection efficiency). Fluorescent reporter protein expression was monitored hourly and replication is presented as both mCherry and ptGFP positive cells at 8 h.p.t. (Figures 2C and S5).

When two WT replicons were co‐transfected, the levels of both ptGFP and mCherry expression indicated replication of both, as expected. The level of mCherry positive cells was slightly lower than that of ptGFP due to the relative sensitivity of detection of these fluorophores by the IncuCyte. The level of ptGFP expression following co‐transfection of the 3B3 mCherry replicon with ptGFP replicons having two or more copies of 3B was similar to WT levels, however, the level of mCherry fluorescence was reduced. These data, therefore, suggest that the mCherry replicon was outcompeted. There was no significant competitive advantage for any specific combination of the 3Bs. Both mCherry and ptGFP signals were reduced (compared to noncompetition levels) when mCherry replicons were co‐transfected with ptGFP replicons containing a single functional 3B, suggesting that they competed equally with each other, thus, reducing the level of expression of each. These data (Figure 2C), together with the data in Figure 1, therefore, suggest that there is a significant replication advantage to possessing multiple copies of 3B compared to having a single copy.

### 3.3 Processing of the 3B junctions is required to maintain primer function

**FIGURE 3:**
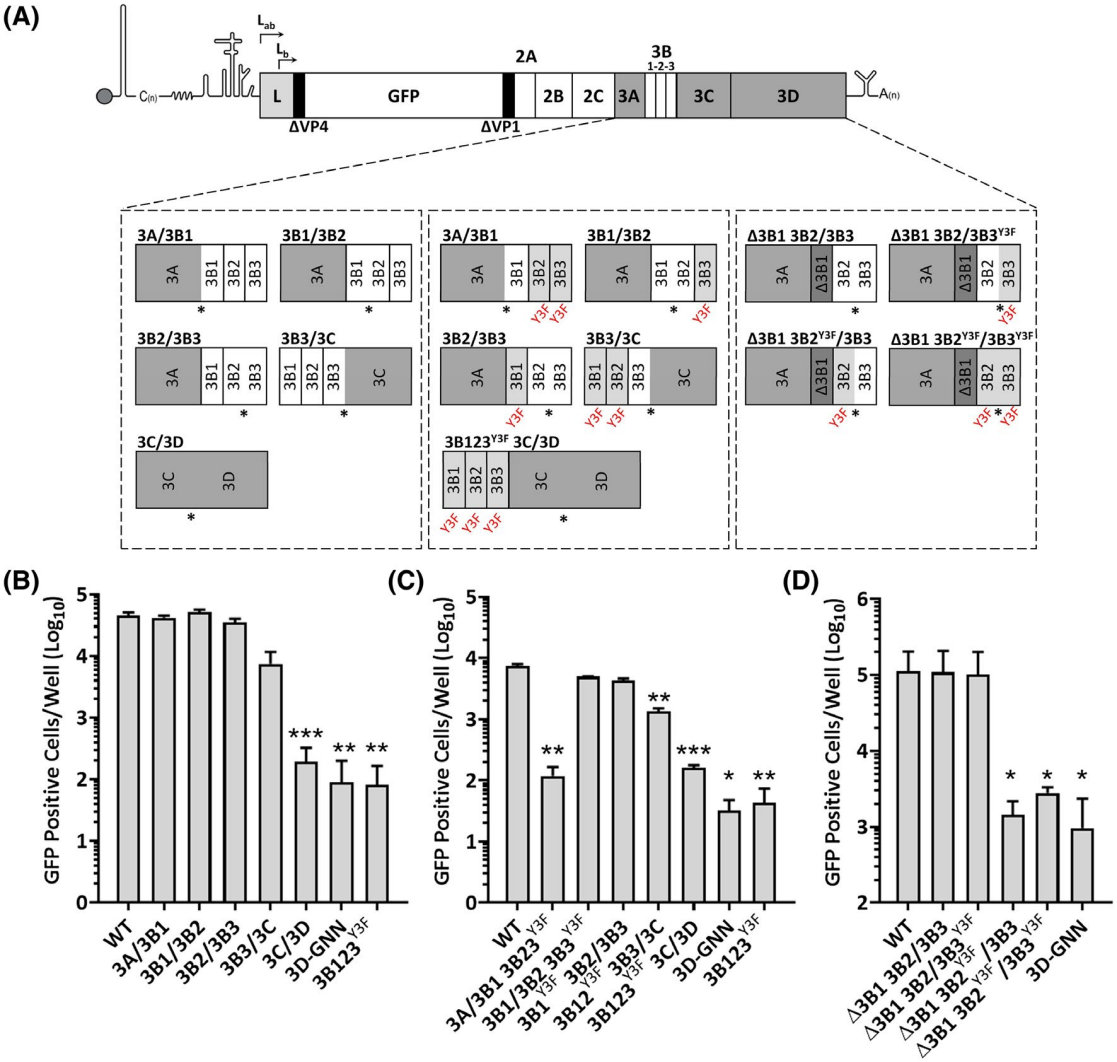
Replication of constructs incorporating gene fusions. A, Schematic of the FMDV replicon. Several fused constructs were designed (Tables 1 and S1) with each 3B fused to its neighbor, including fusions between 3A and 3B1 or 3B3 and 3C. Chimeric constructs were designed to maintain the 3A‐3B1 and 3B3‐3C junctions. The P3 region is expanded for clarity. Fused regions are indicated with asterisks. Replicon constructs were transcribed, and RNA transfected into BHK‐21 cells. Replication was monitored using an IncuCyte. B, Replication of the fused constructs. C, Replication of fusion constructs that incorporate inactivating Y3F substitutions. D, Replication of fusion constructs incorporating both inactivating Y3F substitution and 3B deletions. Figure shows total GFP fluorescence at 8 h.p.t. Baseline represents input translation. Each replicate represents an average of two wells (n = 2 ± S.D., **P < .01, ***P < .001)

Multiple copies of functional 3B appear to offer replication advantages. However, it is not clear that each 3B molecule needs to be released from the polyprotein to provide this advantage. We therefore investigated whether preventing proteolytic release of each molecule from the polyprotein abrogated activity. To do this the sequences at the cleavage sites were mutated to prevent efficient recognition by 3C^pro^. Replicons were generated incorporating alanine substitution to disrupt cleavage boundaries between individual 3B proteins, between 3A and 3B1, and between 3B3 and 3C in different combinations (Figure 3A). The P1 and P2 boundary positions have been shown to be the most critical for 3C^pro^‐mediated proteolysis and introducing alanine residues at these positions in synthetic peptides was shown to abrogate cleavage.^31^ Therefore, these were selected for mutagenesis (except in the context of 3A‐3B1 where the P2 position is already alanine, and therefore, P4 was mutated) (Figure S2). As a control, a replicon was generated in which the 3C‐3D boundary was mutated to block cleavage as we previously showed that preventing the release of functional 3D^pol^ abrogates replication.^17^ We evaluated the consequences of these substitutions on processing using T_N_T assays according to our established protocols.^17^ All the disrupted cleavage boundaries were found to reduce the efficiency of proteolysis and increase the abundance of fused precursors as intended (highlighted in Figure S6). For example, substitution at the 3A‐3B1 boundary increased the abundance of a protein corresponding to the 3A‐3B1 precursor, substitution at the 3B1‐3B2 boundary increased the abundance of a 3A‐3B1‐3B2 precursor, and so forth. In some cases, low level proteolysis at mutated boundaries was still observed, however, this approach was still sufficient to assay the global effect of generating fused protein precursors by cleavage inhibition. After confirming that the substitutions resulted in fused products as anticipated, RNA from the mutated replicons, in addition to WT or 3D‐GNN controls, was transfected into BHK‐21 cells and replication monitored by fluorescent protein expression as before (Figure 3B). As anticipated, substitution of the 3C‐3D junction abrogated replication. There was no significant decrease in replication when any other junction was mutated (Figure 3B).

Next, combinations of 3B^Y3F^ substitutions and cleavage boundary substitutions were designed to create replicons where the only functional 3B proteins were part of a fusion product (fusion products signified using “/,” see Table 1). For example, in the 3A/3B1 3B23^Y3F^ replicon, 3B1 was fused to 3A and both 3B2 and 3B3 were inactivated by Y3F substitution. Therefore, only the 3B1 fusion product could be functional in replication. These constructs were transcribed, RNA transfected into BHK‐21 cells and replication monitored as above. Transfection of some of the constructs resulted in WT levels of replication (Figure 3C), that is, fusion of 3B1 to 3B2 and fusion of 3B2 to 3B3. As these constructs still included one functional 3B molecule, albeit in the context of a fusion protein, these data support previous findings that a single 3B molecule is sufficient for replication. However, in replicons where only 3B1 was unmodified but fused to 3A, replication was greatly reduced, emphasizing the requirement for a “free” N‐terminus of 3B. Fusion of 3B3 to 3C was also deleterious, but much less so.

Further constructs were generated to investigate whether the position of the 3B molecule within a fusion product was important. Replicons were made where 3B1 was deleted (Δ3B1). The 3B2 and 3B3 proteins were then fused together by substitution of the cleavage boundary. Y3F substitutions were introduced to either the N‐terminal 3B (Δ3B1 3B2^Y3F^/3B3) or C‐terminal 3B (Δ3B1 3B2/3B3^Y3F^) or to both as a control (Δ3B1 3B2^Y3F^/3B3^Y3F^). Replicon RNA was then generated and transfected into BHK‐21 cells and monitored for replication as described above. In agreement with the data in Figure 3C, WT levels of replication were recorded when 3B2 and 3B3 were fused, irrespective of whether the 3B3 component of the fusion product was inactivated by Y3F substitution. However, when 3B2 was inactivated, ptGFP expression was similar to the 3D‐GNN negative control, that is, replication was abrogated (Figure 3D). Together, these data suggest that while the 3B protein can still function as a primer for replication with C‐terminal fusions, fusions to the N‐terminus prevent this.

### 3.4 FMDV encodes an optimal number of 3Bs for efficient genome replication

**FIGURE 4:**
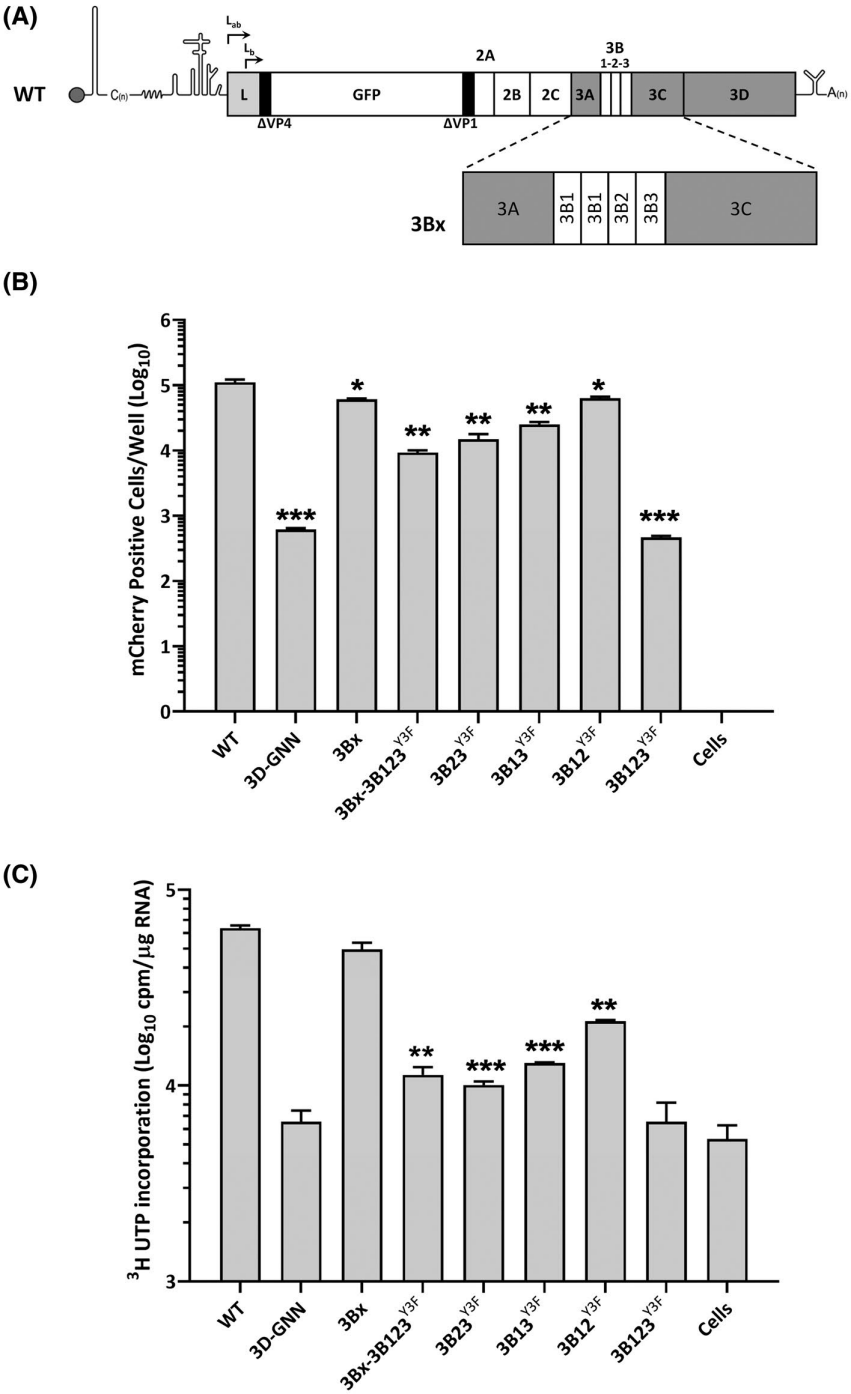
FMDV can tolerate an additional copy of 3B. BHK‐21 cells in duplicate wells of a 6‐well plate were pretreated for 1 hour with actinomycin D at 37°C. Cells were transfected with T7 replicon RNA transcripts, which include a novel 3B (termed 3Bx) as either an additional 3B or as the only functional 3B. Constructs used previously (Figure 1) were also included as controls. At 1 h.p.t., [^3^H]‐U was added, and replication monitored as mCherry expression using an IncuCyte for 8 hours. A, Schematic representation of FMDV WT and 3Bx replicon constructs. B, Total mCherry fluorescence at 8 h.p.t. Each replicate represents an average of two wells (n = 3 ± S.D. *P < .05, **P < .01, ***P < .001). C, At 8 h.p.t., total RNA was extracted and quantified spectrophotometrically. [^3^H]‐U incorporation was measured as scintillation counts per microgram of RNA (n= 3 ± S.D. **P < .01)

Having demonstrated the importance of multiple 3Bs in replication and the requirement for a free N‐terminus, we asked the question whether additional copies of 3B might be tolerated and if so, whether these would offer a further replicative advantage. A mutant construct (termed 3Bx) was designed that encoded an additional copy of 3B1 at the C‐terminus of 3A, while retaining the natural 3A‐3B1 and 3B1‐3B2 cleavage motifs at the new 3A‐3Bx and 3Bx‐3B1 junctions (Figure 4A). Fluorescence was monitored in real time and the levels at 8 h.p.t. shown (Figure 4B). In a parallel experiment, actinomycin‐D pretreated BHK‐21 cells were transfected with T7 RNA transcripts and radiolabeled with [^3^H]‐U. Total RNA was extracted at 8 h.p.t., quantified and purified (Figures 4C and S7). Constructs including previously characterized Y3F mutants (Figure 1) were included as controls and replicated as expected. The 3Bx123 construct which encoded four copies of 3B was replication‐competent, but to a level slightly lower than WT. In the context of a replicon with 3B1, 3B2, and 3B3 inactive, the additional 3B was able to support replication, that is, 3Bx‐3B123^Y3F^ replicated at levels similar to a replicon with just 3B1 (3B23^Y3F^).

### 3.5 Multiple functional copies of 3B are required to rescue defective 3B molecules in *trans*

**FIGURE 5:**
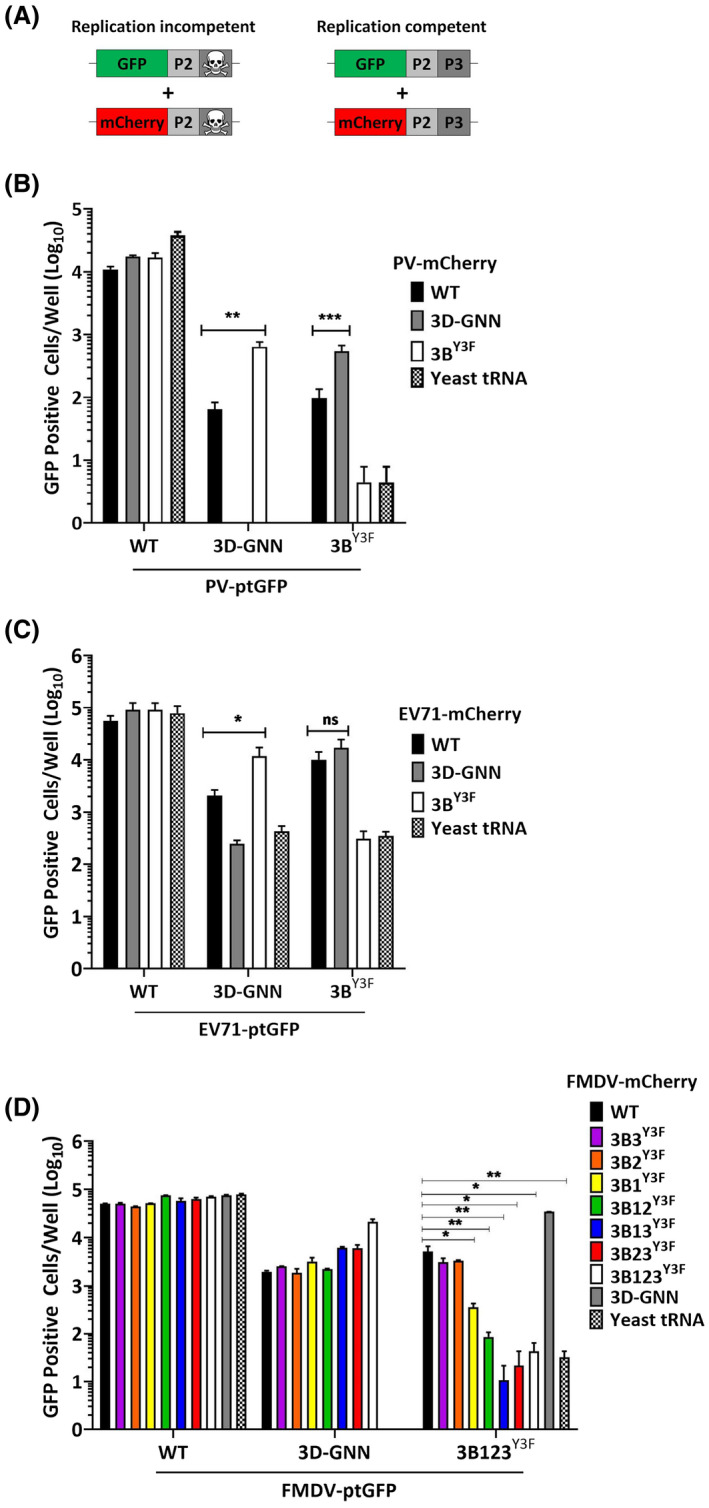
Trans‐complementation by 3B in FMDV differs from enteroviruses. A, Cartoon illustrating trans‐complementation experiment with two replication‐incompetent replicon constructs. Replication‐competent constructs were included as controls. Figures B, C, and D show average fluorescence of duplicate wells at 8 h.p.t. of two replicons co‐transfected into BHK‐21 cells. B, PV‐GNN‐mCherry and PV‐3B^Y3F^‐GFP with controls. Each replicate represents an average of two wells (n = 3 ± S.D., **P < .01, ***P < .001). C, EV71‐GNN‐mCherry and EV71‐3B^Y3F^‐GFP with controls. Each replicate represents an average of two wells (n = 2 ± S.D. *P < .05). D, FMDV‐GNN‐mCherry with GFP replicons encoding one, two, or three active 3Bs. Each replicate represents an average of two wells (n = 3 ± S.D., *P < .05, **P < .01)

It has been documented that some picornaviral proteins can function in *trans* to support replication, as for the enteroviruses PV and enterovirus 71 (EV71).^32^, ^33^ Indeed, we previously showed that some FMDV replicons with replication‐disabling substitutions in the 3D^pol^ active site could be rescued in *trans* by co‐transfection with a second replicon containing a functional 3D^pol^. However, not all substitutions within the 3D^pol^ could be rescued, demonstrating unique *cis* and *trans* activities.^22^ Enteroviruses possess a single copy of 3B and we, therefore, investigated the roles of the multiple copies of FMDV 3Bs in the rescue of replication‐deficient mutant constructs. Design of the complementary rescue experiments employed here is shown in Figure 5A. We also used a PV replicon (serotype 1, termed PV1) and an EV71 replicon (termed EV71) as controls. Replication deficient 3D‐GNN or 3B^Y3F^ substitutions were introduced into WT or mutant ptGFP replicons. RNAs from these constructs were co‐transfected with RNA from WT, 3D‐GNN, or 3B^Y3F^ mCherry replicon constructs (or with yeast tRNA as a transfection control). Replication was monitored by both ptGFP and mCherry expression and data shown at 8 h.p.t. (Figure 5).

All three WT replicons replicated as expected. When WT PV1 or EV71 replicons were co‐transfected with their equivalent 3B^Y3F^ or 3D‐GNN mutant replicons, replication of both was detected (Figure 5B,C). Consistent with our previous data, the FMDV replicons were able to rescue GNN replicons, that is, provide 3D^pol^ *in trans*. However, only FMDV replicon constructs that contained two or more active copies of 3B could restore function to the 3B123^Y3F^ replicon (Figure 5D). Together, our findings suggest that *trans* complementation of enteroviruses such as PV and EV71 requires only one copy of 3B whereas FMDV requires multiple copies of 3B.

## 4 DISCUSSION

The genome of FMDV has several features that are distinct from other members of the *Picornaviridae* family. One of these is the presence of multiple copies of the 3B protein. Indeed, with one exception, all field isolates of FMDV have been shown to encode three copies of the 3B coding region,^11^ however, the reason for the maintenance of these multiple copies is not understood. The uridylylation of the 3B protein is essential for priming genome RNA replication^34^ and a study by King et al^35^ showed that all copies of 3B are present in equal amounts in virion RNA. However, replication could be supported by any one of the three copies.^16^, ^17^ In an in vitro uridylylation assay, Nayak et al demonstrated a preference of synthetic 3B3, over 3B2 and 3B1.^14^ Here, we have employed sub‐genomic replicon constructs incorporating different numbers and combinations of 3B proteins alongside 3B fusions in order to understand the multiplication of this part of the viral genome.

We showed that constructs in which two of the three 3Bs were rendered nonfunctional (by Y3F substitution) but had WT 3B3, replicated to higher levels to those relying solely on WT 3B1 or 3B2, consistent with some of the studies above. However, the effects were subtle and there were slight differences between replication measured via fluorescent reporter expression or via radiolabel incorporation into RNA, possibly due to the different sensitivities of the assays. We also showed that the addition of a fourth 3B coding region (at the C‐terminus of 3A, an area which exhibits a great deal of sequence variation across FMDV isolates) offered no replicative advantage. However, importantly, our studies using combinations of 3Bs, rendered inactive by substitution or by deletion (while maintaining boundary sequences) in competition experiments indicated that a competitive advantage was conferred by possession of more than one 3B.

Constructs were also generated with N‐ or C‐terminal fusions, that is, between 3B molecules and 3A or 3C. By leaving only one 3B functional, it was possible to ascertain whether the fusion could be tolerated for replication. The T_N_T assays demonstrated the expected overall changes in polyprotein boundary cleavage in that substitution of the boundary increased the abundance of the expected precursor. However, in some cases it appears that reducing processing at one site may interfere with the normal cleavage cascade, resulting in enhanced proteolysis at others. It should also be noted that it was not possible to ascertain some small size changes, for example, the fusion of two 3Bs, and some proteolysis of the mutated boundaries was still observed as may be expected from the modest alterations introduced. More dramatic boundary substitutions would have resulted in greater inhibition of proteolysis but this may have resulted in structural changes, which we chose to avoid. However, when taken overall, the data showed that 3Bs with C‐terminal fusions were replication‐competent, whereas N‐terminal fusions were not tolerated, suggesting that the location of the uridylylated tyrosine at position three in 3B is essential for it to function as a primer for RNA synthesis.

Interestingly, previous structural studies of 3B in complex with 3D^pol^ uncovered a tight network of interactions securing the N‐terminus (and Y3 residue) of 3B within the active site cavity of 3D^pol^.^28^ Visual inspection of the structure (PDB‐2D7S) suggests that 3B is tightly “threaded” into the cavity of 3D^pol^ to reach the active site. This potentially explains why the N‐terminal fusions were not tolerated; the 3A‐3B1 fusion would require “threading” the full‐length of 3A through the 3D^pol^ cavity before Y3 could reach its site of action, which is implausible given the narrow dimensions of the cavity (Figure S8). While 3B is smaller and less well ordered than 3A, even for the ∆3B1 3B2^Y3F^/3B3 fusion, the entirety of the nonfunctional 3B2 would need to be threaded through 3D^pol^ to locate 3B3 in place for uridylylation to occur. Given the plethora of interactions that stabilize 3B within the 3D^pol^ cavity,^28^ it is likely that the nonfunctional 3B2 would be held in place by 3D^pol^, preventing any further “threading” needed to position 3B3 correctly. Both of these explanations rely on the assumption that “threading” is one‐way (ie, 3B must enter the 3D^pol^ cavity N‐terminus first to position Y3 correctly); we would argue that this is plausible and may itself explain why Y3 is located so close to the N‐terminus of 3B. This result is also compatible with the suggestion that during the optimal functioning of the replication complex, the active 3B is derived from the N‐terminus of a 3B‐3C(‐3D) precursor and not from the C‐terminus of a 3A‐3B precursor.^36^, ^37^

Sub‐genomic replicons offer the advantage of being able to separate replication from other parts of the virus lifecycle. In this way, we have been able to tease apart the relatively subtle advantages that multiple copies of 3B provide. However, although we have clearly demonstrated that two copies are better than one, the role for a third copy is less clear. It is possible that further competitive passages would be needed for the advantage conferred by three copies to become apparent. It is also possible that the possession of three copies of 3B is important for other aspects of the viral lifecycle, such as immune evasion or virion assembly.

In conclusion, although the single 3B present in the enterovirus replicons used here could rescue a replicon with a defective 3B, rescue of a defective FMDV replicon lacking any functional 3Bs by *trans* complementation required a helper with a minimum of two functional 3Bs. Our previous studies^17^ have also suggested that FMDV can use multiple processing pathways to generate different precursor subsets, and that a 3A‐3B precursor can be used *in trans* to support replication. Together, this suggests FMDV may have evolved two distinct mechanisms of replication, where the extra copies of 3B are nonessential but preferable for optimal *cis* active replication through active 3B‐3C(‐3D) precursors, but the extra copies are necessary to facilitate *trans* complementation via 3A‐3B delivery. This may offer a replicative advantage, thus, providing the selective pressure to maintain multiple copies of the 3B coding region in the genome.

## CONFLICT OF INTEREST

The authors declare no conflict of interests.

## AUTHOR CONTRIBUTIONS

O.O. Adeyemi, J.C. Ward, M.R. Herod, D.J. Rowlands, and N.J. Stonehouse designed the research; O.O. Adeyemi, J.C. Ward, and M.R. Herod designed and conducted the experiments; O.O. Adeyemi, J.C. Ward, J.S. Snowden, and M.R. Herod analyzed data; O.O. Adeyemi, J.C. Ward, and N.J. Stonehouse wrote the manuscript; M.R. Herod, D.J. Rowlands, and N.J. Stonehouse edited the manuscript; D.J. Rowlands, M.R. Herod, and N.J. Stonehouse supervised the project; D.J. Rowlands, M.R. Herod, and N.J. Stonehouse are the guarantors of this work and, as such, had full access to all the data and take responsibility for the integrity of the data and the accuracy of the data analysis.

## Supporting information

Fig S1‐S8

Supplementary Material
