# Supplementary material for: Functional advantages of triplication of the 3B coding region of the FMDV genome
Source: FASEB J. 2020 Nov 23;35(2):e21215. doi: 10.1096/fj.202001473RR (PMC7894486; doi:10.1096/fj.202001473RR)
Supplement: Supplementary file 2 — Supplementary Material [file FSB2-35-e21215-s001.docx]

***Figure S1.* Similarities *of the FMDV 3Bs.*** *Reference sequences of Asia 1 (*[*AQQ72943.1*](https://www.ncbi.nlm.nih.gov/protein/AQQ72943.1)*), Type A (*[*QGQ32933.1*](https://www.ncbi.nlm.nih.gov/protein/QGQ32933.1)*), Type C (*[*AXU41411.1*](https://www.ncbi.nlm.nih.gov/protein/AXU41411.1)*), Type O (*[*YP_009513028.1*](https://www.ncbi.nlm.nih.gov/protein/1464315462)*), SAT 1 (*[*ASV51924.1*](https://www.ncbi.nlm.nih.gov/protein/ASV51924.1)*), SAT 2 (*[*ADI24380.1*](https://www.ncbi.nlm.nih.gov/protein/ADI24380.1)*) and SAT 3 (*[*AAT01793.1*](https://www.ncbi.nlm.nih.gov/protein/AAT01793.1)*) FMDV serotypes were sourced from NCBI. Residue sequences were aligned for homology using the MUSCLE. Figure shows FMDV (A) 3B1 (B) 3B2 and (C) 3B3. Matching residues are represented as dots beneath the corresponding residues of respective 3B. Variable regions are shown as single lettered residues underneath the corresponding positions. (D) A total of 982 sequence isolates that comprise all FMDV serotypes were sourced from NCBI (BLASTp: gb|ADR66170.1|) and aligned using the MUSCLE algorithm of the CLC viewer 7.0. Figure shows the similarity plot of aligned 3B1 and (&) 3B2, 3B1 & 3B3, 3B2 & 3B3 residues (n = 24 ± S.E.M.) and nucleotides (n = 75 ± S.E.M.).*

**Figure S2. Mutated sequences.** *Colour-coded representation of the P3 region of FMDV, highlighting relevant sequences of 3A [black], 3B1 [red], 3B2 [blue], 3B3 [green], 3C [grey] and 3D [black]. Chimeric constructs were designed to maintain the 3A-3B1 and 3B3-3C junctions. P3 boundary regions are symbolised by vertical lines “|”. Partial sequences of chimeric replicon constructs are shown (first and last-5 residues, with exception of 3D where the first 5 residue sequences and the GNN region are shown). Mutations to cleavage boundaries designed to inhibit 3C-induced proteolysis between P3 proteins are shown as red letters with black highlight; Y3F mutations to respective 3B proteins are shown as white letters with black highlight; GNN replication mutation to the 3D polymerase is shown as black letters with cyan highlight. Chimeric constructs were all designed to maintain the 3A-3B1 and 3B3-3C junctions.*

**Figure S3. Sucrose density gradient profiles of ^3^H labelled RNA.** *Extracted RNA was centrifuged through 5% - 25% sucrose gradients (in 100 mM sodium acetate, 0.1% SDS) at 370,000 x g using a SW 55 Ti rotor for 50 minutes at room temperature. Gradient fractions were counted by scintillation for [^3^H]. Graphs show RNA profiles of wt and modified replicons with one, two or three active copies of 3B, as indicated (n = 3 ± S.E.M.).*

**Figure S4. FMDV genome replication over time.** *BHK-21 cells were transfected with T7 RNA transcripts of replicon constructs. These include WT, a polymerase active site mutant (3D-GNN) which was used to indicate input translation level, and replicons where one, two or all the 3Bs have been inactivated by Y3F substitution (Table 1). Replication was monitored as hourly expression of mCherry (n = 3 ± S.D., *P<0.05).*

**Figure S5. Controls for the FMDV 3B competition assays.** *Equimolar concentrations of FMDV replicon T7 RNA transcripts with one, two or three copies of 3B were co-transfected with yeast tRNA into BHK cells. Figures show maximum fluorescence positive cells at 8 hours post transfection. Each data set represents an average of two wells (n = 3 ± S.D.).*

**Figure S6. Cleavage boundary mutations that prevent processing and generate fusion precursors.** *Plasmids expressing wild-type FMDV P3 or P3 polyproteins with a fused boundary (indicated by /) were used to assemble coupled transcription/translation reactions with [^35^S] labelled methionine. Reactions were incubated for 40 minutes before being chased with excess unlabelled methionine/cysteine. Samples were taken at 30-minute intervals, stopped by addition of 2x Laemmli buffer, separated by 12% SDS-PAGE and visualised by autoradiography. The positions of FMDV proteins and precursors are labelled with arrows. The fusion precursors are indicated by red asterisks.*

**Figure S7. Sucrose density gradient profiles of RNA.** *RNA extracts were centrifuged through 5% - 25% sucrose gradients (in 100 mM sodium acetate, 0.1% SDS) at 370,000 x g in a SW 55 Ti rotor for 50 minutes at room temperature. Gradient fractions were counted by scintillation for [^3^H]. Graph shows RNA purification profiles of WT and constructs with up to four copies of 3B, as indicated (n = 3 ± S.E.M.).*

**Figure S8. Structural arrangement of FMDV 3B in complex with 3D (PDB-2D7S) (Ferrer-Orta *et al.*, 2006).** *(A) Atomic coordinates of 3B (magenta) in complex with 3D (green), with the critical Y3 residue of 3B highlighted in cyan. (B) FMDV 3B shown within the 3D cavity. 3D is represented in molecular surface view. Inset shows an enlarged view of the boxed region.*
